# Discrimination of Glutopeak Test and Mixograph Parameters for Evaluation of Wheat Flour Supplemented with Hazelnut Skin, Cross-Linked Starch, and Oxidized Starch

**DOI:** 10.3390/foods12020328

**Published:** 2023-01-10

**Authors:** Yusuf Durmus, Munir Anil, Senay Simsek

**Affiliations:** 1 Department of Gastronomy and Culinary Arts, School of Applied Sciences, Artvin Coruh University, Artvin 08100, Turkey; 2 Department of Food Engineering, Ondokuzmayis University, Samsun 55000, Turkey; 3 Department of Food Science, Whistler Center for Carbohydrate Research, Purdue University, West Lafayette, IN 47907, USA

**Keywords:** hazelnut skin, Glutopeak, modified starch, dough property

## Abstract

Hazelnut skin is a by-product produced from hazelnut processing. It can be used as a nutritional supplement due to its high nutrient values. The purpose of this study was to evaluate the dough properties of refined flour (RF) with the addition of hazelnut skin (HS), cross-linked starch (CS), and oxidized starch (OS). Principal component analysis showed a positive correlation between maximum torque, torque after maximum, and aggregation energy. Usage of 10% HS significantly (*p* < 0.05) decreased the mixograph MID line peak value indicating a weaker dough. Random forest (RFT) was conducted to classify the samples and to determine the importance levels of the analysis parameters. According to the results, AE and mixograph MID line peak values were the most discriminant parameters to distinguish the samples into groups. High-level HS alone caused undesirable effects on the dough quality, yet the addition of modified starches could be used to compensate for the undesirable effects. When used together, the interaction between hazelnut skin and modified starches should be considered. Glutopeak provides a means for assessing the impacts of additives such as hazelnut skin or modified starches on flour functionality.

## 1. Introduction

In addition to the products with technological advantages, products with increased nutritional value and the recycling of waste and by-products are of great interest. Hazelnut skin is a by-product obtained after roasting during the processing of hazelnut and is very rich in phenolic, antioxidant, and dietary fiber content [1]. Due to their benefits for human health, dietary fibers are becoming more popular as food ingredients in baked goods. When nutritional fibers such as wheat bran are used for bread-making, the result is a darker color and smaller loaf sizes. Wheat bran also alters the rheology of dough, increases water absorption, and makes processing dough more challenging [2]. Anil [3] came to the conclusion that adding 5–10% of hazelnut skin to bread would improve the amount of nutritional fiber while retaining the bread’s quality and sensory appeal. Unfortunately, the use of dietary fibers in bread production causes a decrease in the rheological properties of the dough, undesirable color, and lower loaf volumes [4]. Due to the fact that hazelnut skin is rich in natural phenolic antioxidants, some researchers have attempted to use hazelnut skin in bread formulations; however, they obtained low loaf volumes and sensory properties [3,5]. Modified starches can be used to address the challenges of incorporating hazelnut skin into bread production.

With the modification of the starch, some technological expansions are achieved by increasing the functionality of native starch. Bonds between hydroxyl groups are responsible for the production of cross-linked starch in the starch structure. Since the cross-linked starch has low digestibility, it is also considered a dietary fiber and is used because of its effects, such as increased viscosity and stability in foods [6]. For example, it has been found that mixograph mix time increased gradually with the increasing addition of cross-linked starch [7]. Oxidized starches, as another type of modified starch, are formed when the carboxyl and carbonyl groups of starch depolarize. Although many agents are used as oxidizing agents, sodium hypochlorite is widely used. It was demonstrated that adding 3% oxidized potato starch to flour significantly increases the resistance to extension of doughs [8]. Chittrakorn [9] found that mixograph peak time increased with the ozone treatment of soft wheat flour. Sui et al. [10] reported that ozone treatment of wheat flour from 15 s to 5 min slightly affected the peak time and ranged from 1.28 to 2.48 min.

The purpose of this study was to investigate the individual and combined effects of hazelnut skin, cross-linked starch, and oxidized starch on dough quality. It was also aimed to compare the glutopeak test and mixograph parameters and to reveal the relative relevance of the parameters with principal component analysis (PCA) and random forest (RFT).

## 2. Materials and Methods

### 2.1. Materials

The North Dakota State Mill supplied commercial hard red spring wheat flour free of oxidizing agents and other ingredients (Grand Forks, ND, USA). Hazelnut skin (perisperm from *Corylus avellana* L.) was obtained from a hazelnut processing plant in Ordu, Turkey. Hazelnut skin was ground in a laboratory grinder and sifted through sieves with a mesh size range of 0.50 to 0.85 mm. Both modified corn flour oxidized starch (sodium hypochlorite, NaClO) and modified corn flour cross-linked starch (sodium trimetaphosphate, Na_3_P_3_O_9_) were obtained from Pendik Nisasta (Istanbul, Turkey). Hazelnut skin at 5% and 10% flour replacement was used along with 3% and 5% of modified starches on a flour basis. Each sample was prepared in triplicate.

### 2.2. Dough Quality

The Glutopeak test was conducted using a Glutopeak device (Brabender, Duisburg, Germany), according to Chandi and Seetharaman [11]. To maintain a constant liquid-to-solid ratio, an aliquot of 8.5 g of flour was mixed with 9.5 g of distilled water. Both the water and flour weights were scaled to 14% of the moisture content of the flour. The test was performed for 7 min with the paddle rotating at 1900 rpm. The primary metrics measured were the maximum torque (MT), which represents the peak as the gluten aggregates, the peak maximum time (PMT), which represents the period of time before the torque starts to decline as the gluten starts to degrade, the torque after maximum (TAM), which represents the torque 15 s after PMT, and the area under the peak (or energy), which was determined by integrating the curve and expressed in arbitrary units (AU). The Glutopeak software, version 1.1.0, was used to calculate all parameters. The measurements were carried out in duplicate. Dough mixing properties were determined using a mixograph with the Mixsmart software version 3.8 according to AACCI method 54-40.02 [12].

### 2.3. Principal Component Analysis (PCA) and Random Forest (RFT)

The Glutopeak and mixograph results of samples were analyzed through PCA to establish the linear relationships between the samples and between the results. The data were standardized and decomposed into principal components (PCs), and the biplot was drawn using the first two PCs [13].

RFT is an ensemble machine-learning model that can be applied to both classification and regression operations. It contains multiple decision tree models. Each tree is built using a unique “bag” of bootstrap samples from the training set. When separating the nodes, the best predictor variable from a group of predictors randomly chosen for each node is taken into consideration. The remaining data are utilized as a test set for this particular tree because it is regarded as being “out of the bag.” It offers information on the variable importance rate [14]. A machine learning model was built using the random forest (RFT) algorithm to distinguish the categorized variables on the basis of the Gini index for each node split impurity. Five trees were used to build the model. Two and one samples were utilized, respectively, as the minimal number needed to divide an internal node and a leaf node. The nodes were expanded until each leaf included no more than two samples. The model was validated using the train–test split method; 20% of the data were used for testing, and the remaining 80% were used for training. The classification accuracy for both the training and the test models was 100%. The model was then utilized to perform variable importance measurement in order to determine the relative relevance of each parameter used in the glutopeak test and mixograph. Python programming language (version 3.9) was used to build the PCA and RFT models.

### 2.4. Statistical Analysis

The analysis of variance (ANOVA) was carried out using statistical analysis software (SAS v.9.4). For mean separation, the least significant difference (LSD) was applied.

## 3. Results and Discussion

### 3.1. Glutopeak Test

The Glutopeak test can evaluate the gluten aggregation behavior of whole wheat and refined flours. In general, flour with poor technological quality is characterized by a rapidly developing consistency and a sharp peak, followed by the rapid breakdown of gluten. In contrast, strong flour slowly develops the dough consistency and requires more time to achieve the peak consistency [15]. Glutopeak test parameters for the different flour blends are shown in Table 1. Hazelnut skin and modified starch increased the time required for peak maximum, which was more pronounced in samples containing both hazelnut skin and modified starch (Table 1). Cross-linked starch did not significantly (*p* < 0.05) affect the PMT, but oxidized starch did cause an increase in PMT (Table 1). According to Sissons [16], PMT is a suitable alternative parameter for both mixograph and gluten index for evaluating gluten strength. Hazelnut skin had a decreasing effect on MT and was significant (*p* < 0.05) with the use of 10% hazelnut skin (Table 1). Samples containing 10% hazelnut skin had low TAM values compared to 0% and 5% hazelnut skin. Both modified starches showed a reducing effect on TAM and a significant (*p* < 0.05) decrease in the TAM of dough containing hazelnut skin. It was observed that AE showed some variation between samples with hazelnut skin (Table 1).

The Glutopeak test has the potential to be utilized as a quick tool for testing the quality of flour, but comparing the findings is challenging due to varying testing parameters such as mixing speed and flour to water ratio used in the literature [17]. Marti et al. [18] discovered that the Glutopeak test AE is a better indicator of semolina gluten strength than MT and PMT, according to correlations with gluten index and alveograph W value. The AE results showed a high correlation with MT and TAM results (Table 1). Marti et al. [15] also detected that there was a high negative correlation between AE and farinograph water absorption values. Cardone et al. [19] indicated that, whereas MT significantly (*p* < 0.05) increased with the addition of wheat bran, PMT and AE significantly (*p* < 0.05) decreased. On the other hand, another study reported that the addition of bran to flour increased MT, while PMT and AE significantly decreased [20]. Although AE showed a similar trend with our study, different results were obtained in PMT and MT, which may be due to the different glutopeak test conditions. In addition, high phenolic content of hazelnut skin may have caused the formation of disulfide bonds in gluten and strengthening of gluten [21]. Wang et al. [22] used whole wheat flour in their study and found that the PMT decreased significantly with the decrease in particle size, and the MT increased significantly. MT was reported to have a moderate positive correlation with bread volume [23]. Rakita et al. [24] stated that MT and AE showed a high positive correlation between bread specific volume and gluten strength. It was reported that flours with low PMT, but high MT and AE may result in breads with low crumb hardness and chewiness [24]. High values of the AE indicate that gluten requires high energy and a long time for aggregation. In addition, samples with high AE are more stable during prolonged mixing. It was observed that AE significantly (*p* < 0.05) decreased with the addition of modified starches, which was more pronounced in oxidized starch. On the other hand, a PMT value of 70.5 s was observed when 10% hazelnut skin was used alone. When 5% cross-linked starch and 5% oxidized starch were added, PMT was 106.0 and 108.5, respectively. It was observed that MT, TAM, and AE values obtained with 10% hazelnut skin decreased statistically significantly (*p* < 0.05) with the addition of 5% modified starches. Overall, the findings of the Glutopeak test were generally worsened by the addition of 10% hazelnut skin, and cross-linked starch performed better than oxidized starch. Accordingly, it is clear that there is an interaction between hazelnut skin and modified starches. The high dietary fiber content of the hazelnut skin causes its water binding capacity to be high. This causes the starch granules to swell less due to a lack of water [25]. The fat content in the hazelnut skin used in this study was 26.68% [2]. Emulsifiers and oil are known to interact with starch granules and affect gelatinization and retrogradation [2]. The oil and dietary fiber present in the hazelnut skin may have caused the glutopeak parameters of starch to change significantly. Another reason for the interaction that may occur between hazelnut skin and starch is the phenolic compounds in the hazelnut skin. It was reported that the swelling and solubility ability of starch decreases as a result of interactions with phenolic compounds [21,26]. Phenolic compounds were reported to strengthen gluten by forming disulfide bonds. It was also stated that starch can absorb phenolic compounds due to its porous structure, and hydrogen bonds may form between starch and phenolics [21,26]. According to Demirkesen et al. [27], starch–starch interactions are prevented by the hydrogen bonds that develop between dietary fiber and starch.

**Figure 1:**
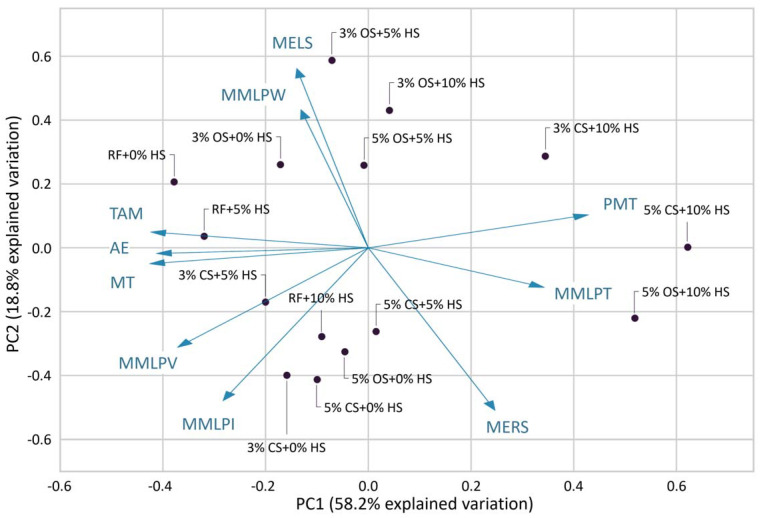
Biplot of principal component analysis (PCA) illustrating the overall variation among the flour samples with hazelnut skin, as well as cross-linked and oxidized starches. RF = refined flour, HS = hazelnut skin, CS = crosslinked starch, OS = oxidized starch, PMT = peak maximum time, MT = maximum torque, TAM = torque after maximum, AE = aggregation energy, MELS = mixograph envelope left slope, MERS = mixograph envelope right slope, MMLPT = mixograph MID line peak time, MMLPV = mixograph MID line peak value, MMLPW = mixograph MID line peak width, MMLPI = mixograph MID line peak integral.

The PCA biplot provides an overview of the similarities and differences among the flour samples and the interrelationships among the measured properties (Figure 1). The distance between the locations of any two samples on the biplot is directly proportional to the degree of similarity or difference. PC1 and PC2 explained 58.2% and 18.8% of the data variation. Together, the first two principal components represented 77.0% of the total variability. The 3% CS + 10% HS, 5% CS + 10% HS, and 5% OS + 10% HS samples were located on the right side of the plot with high positive PC1 values, different from the other samples. This is most likely due to the dough strength impacted by the 10% HS content in each sample. RF samples were linearly located on the biplot according to the addition of hazelnut skin. With the addition of 3% cross-linked starch to flour (3% CS + 0% HS), the Glutopeak test and mixograph properties changed greatly, while with the addition of 5% cross-linked starch (5% CS + 0% HS), no further changes occurred. The increased levels of hazelnut skin for both types of starch greatly altered the properties of flour, which may have been caused by the interaction between starches and hazelnut skin.

**Figure 2:**
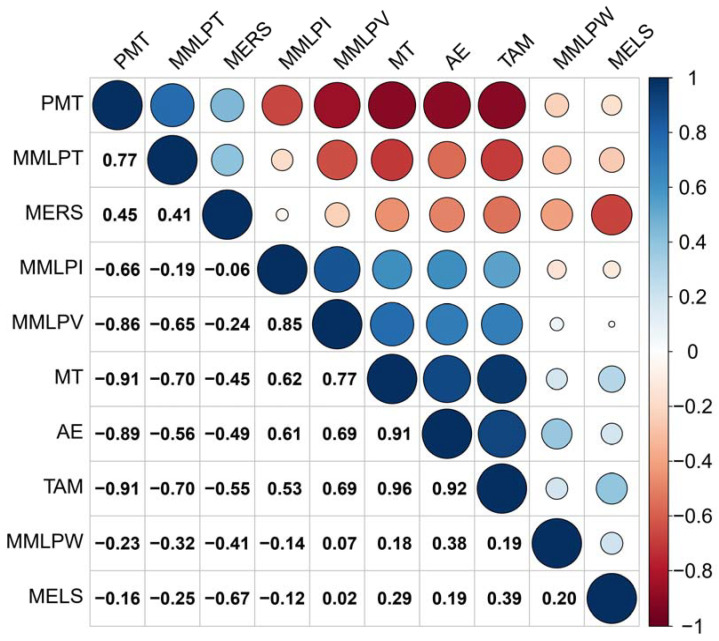
Correlation results for the glutopeak test and mixograph results. PMT = peak maximum time, MT = maximum torque, TAM = torque after maximum, AE = aggregation energy, MELS = mixograph envelope left slope, MERS = mixograph envelope right slope, MMLPT = mixograph MID line peak time, MMLPV = mixograph MID line peak value, MMLPW = mixograph MID line peak width, MMLPI = mixograph MID line peak integral.

The relationships between the Glutopeak and mixograph features were revealed using the biplot of the principal component analysis (PCA) (Figure 1). A positive correlation exists between properties with curves that are close to one another on the plot, and a negative correlation exists between properties with curves that run in opposite directions. The inferences of a positive relationship among TAM, AE, and MT in the glutopeak test parameters, in addition to the PMT having a negative correlation with these, can be made in Figure 1 and Figure 2. Güçbilmez et al. [23] reported a moderate negative correlation between PMT and AE (−0.5318) which is consistent with this study. Sissons [16] found a strong relationship between PMT and mixograph MID line peak time (MMLPT) as indicators of gluten strength. Similarly, PMT and MMLPT are located close to each other on the biplot shown in Figure 1.

**Figure 3:**
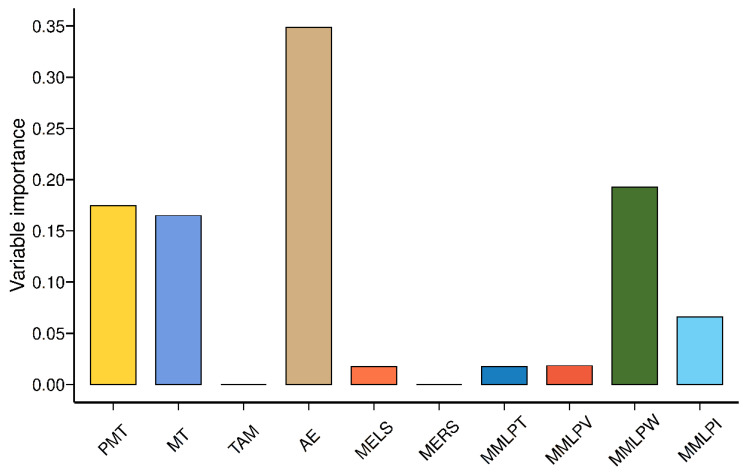
Variable importance measurement based on the RFT model for the Glutopeak test and mixograph results according to the addition of modified starches. PMT = peak maximum time, MT = maximum torque, TAM = torque after maximum, AE = aggregation energy, MELS = mixograph envelope left slope, MERS = mixograph envelope right slope, MMLPT = mixograph MID line peak time, MMLPV = mixograph MID line peak value, MMLPW = mixograph MID line peak width, MMLPI = mixograph MID line peak integral.

**Figure 4:**
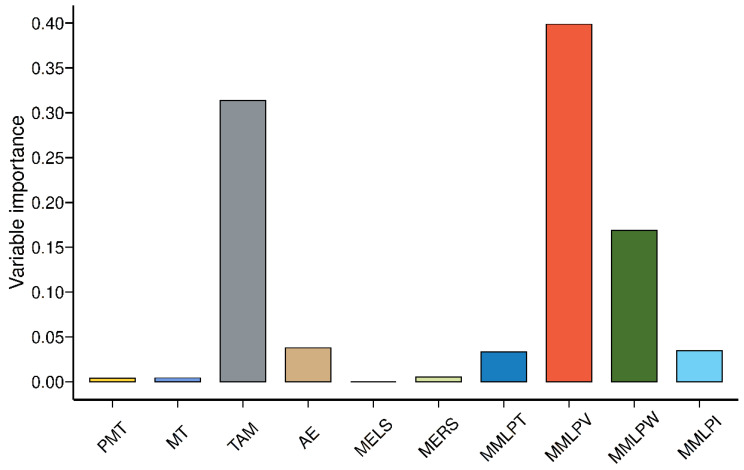
Variable importance measurement based on the RFT model for the Glutopeak test and mixograph results according to the addition of hazelnut skin. PMT = peak maximum time, MT = maximum torque, TAM = torque after maximum, AE = aggregation energy, MELS = mixograph envelope left slope, MERS = mixograph envelope right slope, MMLPT = mixograph MID line peak time, MMLPV = mixograph MID line peak value, MMLPW = mixograph MID line peak width, MMLPI = mixograph MID line peak integral.

Random forest (RFT) is an ensemble tree-building technique in which data are divided into several consecutive class subsets, depending on whether the variable is significantly related to the response variable. In RFT models, many classification and regression trees (CARTs) are built and ensembled to obtain more robust and reliable results. Multiple CARTs are generated with RFT, and these are used by combining them to get a more accurate and stable prediction. RFT algorithm is a machine learning method capable of feature selection [14]. In this study, we used RFT as a feature selection method to differentiate the importance levels of analysis parameters. Two RFT models were built in this study, and the response variables in our case were modified starch addition (Figure 3) and hazelnut skin addition (Figure 4). RF samples were highly different from cross-linked and oxidized starch samples (Figure 3). The variable importance measurement for RFT can easily discriminate to what extent features are important for categorization. Variable importance measurements were performed using each RFT model. AE was the most important parameter to distinguish the samples (Figure 3). TAM results were not selected as part of the model, suggesting that they did not add to the discriminative value of the RFT. The absence of TAM was probably due to its high correlation with AE, as shown in Figure 2. Since RFT is a feature selection method, it decreases the importance degree of one or more highly correlated variables to avoid overfitting. For hazelnut skin, TAM had the highest importance level among the Glutopeak test parameters for separating the samples (Figure 4).

### 3.2. Mixograph Analysis

Mixograph results for the different flour blends are summarized in Table 2. Mixograph can be used to monitor the development of the gluten network during dough mixing. Mixograph results are widely used to screen for gluten strength and dough mixing properties of flour [28]. Generally, mixograph MID line peak time (MMLPT), mixograph MID line peak value (MMLPV), and mixograph MID line peak integral (MMLPI) were all positively correlated to dough strength. Mixograph envelope left (MELS) and right (MERS) slopes correlated with resistance to extension, and mixograph MID line peak width (MMLPW) was positively correlated with over mixing tolerance [29]. MMLPT indicates the time required for the development of dough, which ranged between 3.55 and 5.15 min for different flour blends (Table 2).

It was determined that hazelnut skin remarkably extended the required time for mixing. These results indicate that MMLPT for optimum dough development increased as the cross-linked starch content increased. This may be due to the slow hydration of the cross-linked starch. The same amount of water was used for each mixograph test; therefore, water absorption capacity impacts mixograph parameters. According to mixograph properties, dough with a larger peak width, longer mixing time, and lower breakdown indicates a stable and strong dough. The MMLPV and MMLPW tended to decrease at the higher levels of modified starch, indicating a weaker dough. While adding 3% oxidized starch to flour did not significantly (*p* > 0.05) affect the mixograph properties, MMLPW was significantly (*p* < 0.05) reduced by increasing the oxidized starch level to 5% (Table 2). MELS and MERS did not change significantly (*p* > 0.05) with the addition of hazelnut skin and modified starches. This partially corroborates the finding of Al-Foudari et al. [30], who reported that MMLPT and MERS did not change significantly with the addition of 10% and 20% wheat bran to flour, while MELS decreased significantly. Chen et al. [31] stated that MMLPV decreased with the addition of wheat bran to flour, and this decrease was more pronounced with the increase in bran particle size. This result is in accordance with the MMLPV results in this study. MMLPV and MMLPW were shown to have a moderate positive correlation with bread loaf volume [32]. MMLPT and MMLPV were reported to have a positive correlation with surface smoothness and crumb structure in Chinese steamed breads [33]. Overall, oxidized starch alone typically produced superior results to cross-linked starch, whereas the usage of hazelnut skin with oxidized starch improved the rheology of the dough. Due to its high dietary fiber and antioxidant content, hazelnut skin can enhance the nutritional value of bread but lowers the bread’s quality. According to Gómez et al. [34], the addition of dietary fiber in bread decreased crumb firmness due to the ability to bind water and may interact with starch to slow down starch retrogradation.

The biplot of principal component analysis (PCA) is shown in Figure 1. There was a positive correlation between MELS and MMLPW, as well as a strong negative relationship between them and MERS. Samples with oxidized starch were mainly located on the upper side of the plot. MELS and MMLPW values of these samples were fairly high (Table 2) Here, 5% CS + 10% HS and 5% OS + 10% HS were the two samples showing mixograph patterns that differed from control flour (RF + 0% HS). MMLPV results in this study had a positive correlation with MT, TAM, and AE values measured by the Glutopeak test.

The RFT for modified starch addition is shown in Figure 3. The most discriminating parameter was MMLPV for the hazelnut skin addition (Figure 4). MERS did not have any effect on differentiating the modified starch samples and had a very limited effect on the hazelnut skin addition. MMLPW was a fairly important parameter to differentiate the samples for both modified starch and hazelnut skin addition. MMLPV had the highest importance level, while MMLPT and MMLPI had similar importance levels (Figure 4).

## 4. Conclusions

This research demonstrated that 10% hazelnut skin had significant effects on Glutopeak test properties and mixograph characteristics in wheat flour blends. As hazelnut skin concentration increased, MT and TAM values gradually decreased. The addition of 5% hazelnut skin had almost no effect on glutopeak test results. However, the fact that the addition of 5% hazelnut skin caused significant changes in the samples containing modified starch indicates the existence of an interaction between hazelnut skin and modified starches. Glutopeak test results were negatively affected by the addition of 10% hazelnut skin. It was observed that cross-linked starch had better results than oxidized starch, and 3% was appropriate to compensate for the unfavorable effects of hazelnut skin. PCA showed that there were highly positive correlations among TAM, AE, and MT, between MELS and MMLPV, and between PMT and MMLPT results of the Glutopeak test and mixograph. Similar results were obtained between the use of oxidized starch at 3% and 5% and the use of cross-linked starch in the same proportions in terms of effects on dough strength. The mixograph findings revealed that oxidized starch was favorable compared to cross-linked starch, while 3% utilization yielded better results for both starches. The use of 5% hazelnut skin provided appropriate results; however, 10% hazelnut skin led to a decline in dough quality. According to RFT results, in order to observe the effects of modified starch addition on flour, the most important parameters were determined as AE, PMT, MT, and MMLPW, whereas TAM, MMLPV, and MMLPW parameters were decisive for the addition of hazelnut skin. Overall, the use of hazelnut skin in flour formulation at certain levels is appropriate in terms of dough quality, and the interaction capability between hazelnut skin and modified starches should be taken into consideration when used together.

## Figures and Tables

**Table 1:** Glutopeak test results of dough prepared with hazelnut skin and modified starches.

	Modified Starch	Hazelnut Skin	PMT	MT	TAM	AE
	(%)	(%)	(sec.)	(GPU)	(GPU)	(AU)
Refined flour	0	0	58.5 h	75.0 a	55.5 ab	1741.6 ab
Refined flour	0	5	58.5 h	74.0 ab	56.0 a	1748.2 a
Refined flour	0	10	70.5 cdef	70.0 cde	51.5 ef	1697.9 abc
Cross-linked	3	0	65.5 efgh	72.5 abc	54 abcd	1676.2 cd
Cross-linked	3	5	59.5 gh	72.0 bcd	54.5 abc	1696 bcd
Cross-linked	3	10	95.0 b	60.5 f	45.0 g	1497.1 g
Cross-linked	5	0	62.5 fgh	72.5 abc	52.5 cdef	1609.9 ef
Cross-linked	5	5	74.5 cde	68.5 e	52.0 def	1579.4 f
Cross-linked	5	10	106.0 a	56.5 g	42.0 h	1409.2 h
Oxidized	3	0	68.5 defg	69.5 de	51.5 ef	1588.1 f
Oxidized	3	5	79.5 c	71.5 bcd	54.5 abc	1623.0 ef
Oxidized	3	10	77.5 cd	69.5 de	51.5 ef	1645.1 de
Oxidized	5	0	78.0 c	72.0 bcd	50.5 f	1599.6 ef
Oxidized	5	5	79.5 c	71.0 cde	53.5 bcde	1619.6 ef
Oxidized	5	10	108.5 a	61.5 f	44.5 g	1494.0 g

PMT = peak maximum time, MT = maximum torque, TAM = torque after maximum, AE = aggregation energy, GPU = Glutopeak unit, AU = arbitrary units. Values in the same column with the same letter are not significantly (*p* < 0.05) different.

**Table 2:** Mixograph results of dough prepared with hazelnut skin and modified starches.

	Modified Starch	Hazelnut Skin	MELS	MERS	MMLPT	MMLPV	MMLPW	MMLPI
	(%)	(%)	(%/min)	(%/min)	(min)	(%)	(%)	(% torque·min)
Refined flour	0	0	23.0 a	−10.3 a	3.7 cd	68.9 a	24.4 ab	196.1 ab
Refined flour	0	5	30.4 a	−10.0 a	4.7 abcd	63.9 ab	17.0 abcde	240.8 a
Refined flour	0	10	13.8 a	−5.6 a	4.5 abcd	60.7 bc	22.8 abcd	221.2 a
Cross-linked	3	0	20.0 a	−5.1 a	4.2 bcd	65.0 ab	14.4 de	224.3 a
Cross-linked	3	5	17.0 a	−6.5 a	4.0 bcd	61.2 bc	19.9 abcde	197.2 ab
Cross-linked	3	10	21.6 a	−9.0 a	4.9 abc	39.3 ef	18.4 abcde	136.4 bcd
Cross-linked	5	0	13.6 a	−5.3 a	3.6 d	62.8 abc	12.9 e	182.8 abc
Cross-linked	5	5	22.8 a	−6.9 a	4.9 abc	57.1 c	12.9 e	225.6 a
Cross-linked	5	10	19.5 a	−3.5 a	5.2 ab	33.9 f	15.5 cde	111.7 d
Oxidized	3	0	30.4 a	−8.0 a	3.6 d	68.5 a	23.5 abc	187.1 abc
Oxidized	3	5	33.2 a	−10.5 a	4.0 bcd	43.6 de	17.8 abcde	127.20 cd
Oxidized	3	10	25.4 a	−7.2 a	4.6 abcd	39.0 ef	25.7 a	133.0 cd
Oxidized	5	0	23.2 a	−5.8 a	4.7 abcd	65.8 ab	15.4 cde	239.0 a
Oxidized	5	5	31.3 a	−6.4 a	4.2 bcd	46.2 d	17.3 abcde	147.7 bcd
Oxidized	5	10	13.7 a	−3.7 a	5.5 a	35.5 f	15.9 bcde	151.7 bcd

MELS = mixograph envelope left slope, MERS = mixograph envelope right slope, MMLPT = mixograph MID line peak time, MMLPV = mixograph MID line peak value, MMLPW = mixograph MID line peak width, MMLPI = mixograph MID line peak integral. Values in the same column with the same letter are not significantly (*p* < 0.05) different.

## Data Availability

Data is contained within the article.

## References

[1] Alasalvar C., Karamac M., Kosinska A., Rybarczyk A., Shahidi F., Amarowicz R. (2009). Antioxidant activity of hazelnut skin phenolics. J. Agric. Food Chem..

[2] Durmus Y., Anil M., Simsek S. (2021). Effects of hazelnut skin, cross-linked starch, and oxidized starch on wheat flour and dough quality. J. Food Process. Preserv..

[3] Anil M. (2007). Using of hazelnut testa as a source of dietary fiber in bread-making. J. Food Eng..

[4] Han W., Ma S., Li L., Zheng X., Wang X. (2019). Impact of wheat bran dietary fiber on gluten and gluten-starch microstructure formation in dough. Food Hydrocoll..

[5] Velioglu S.D., Güner K.G., Velioğlu H.M., Çelikyurt G. (2017). The use of hazelnut testa in bakery products. J. Tekirdag Agric. Fac..

[6] Singh J., Kaur L., McCarthy O. (2007). Factors influencing the physico-chemical, morphological, thermal and rheological properties of some chemically modified starches for food applications—A review. Food Hydrocoll..

[7] Miller R.A., Bianchi E. (2017). Effect of RS4 resistant starch on dietary fiber content of white pan bread. Cereal Chem. J..

[8] Xinwen Z., Haizhou D., Chuanfu L., Yuqiu G., Xiao L. (2014). Effects of Potato Oxidized Starch on Dough Characteristics and Quality of Steamed bread. J. Chin. Cereals Oils Assoc..

[9] Chittrakorn S. (2008). Use of Ozone as an Alternative to Chlorine for Treatment of Soft Wheat Flours.

[10] Sui Z., Yao T., Zhong J., Li Y., Kong X., Ai L. (2016). Ozonation treatment improves properties of wheat flour and the baking quality of cake. Philipp. Agric. Sci..

[11] Chandi G.K., Seetharaman K. (2012). Optimization of gluten peak tester: A statistical approach. J. Food Qual..

[12] Cereals and Grains Association (2009). AACC International Approved Methods of Analysis.

[13] Jolliffe I.T., Cadima J. (2016). Principal component analysis: A review and recent developments. Philos. Trans. R. Soc. A Math. Phys. Eng. Sci..

[14] Breiman L. (2001). Random forests. Mach. Learn..

[15] Marti A., Augst E., Cox S., Koehler P. (2015). Correlations between gluten aggregation properties defined by the GlutoPeak test and content of quality-related protein fractions of winter wheat flour. J. Cereal Sci..

[16] Sissons M. (2016). GlutoPeak: A Breeding Tool for Screening Dough Properties of Durum Wheat Semolina. Cereal Chem. J..

[17] Wang K., Dupuis B., Fu B.X. (2017). Gluten Aggregation Behavior in High-Shear-Based GlutoPeak Test: Impact of Flour Water Absorption and Strength. Cereal Chem..

[18] Marti A., Cecchini C., D’Egidio M.G., Dreisoerner J., Pagani M.A. (2014). Characterization of Durum Wheat Semolina by Means of a Rapid Shear-Based Method. Cereal Chem..

[19] Cardone G., D’Incecco P., Casiraghi M.C., Marti A. (2020). Exploiting milling by-products in bread-making: The case of sprouted wheat. Foods.

[20] Wang K., Sangha J., Cuthbert R., Fu B.X. (2021). Effectiveness and biochemical basis of wholemeal GlutoPeak test in predicting water absorption and gluten strength of Canadian hard red spring wheat. Cereal Chem..

[21] Girard A.L., Castell-Perez M.E., Bean S.R., Adrianos S.L., Awika J.M. (2016). Effect of condensed tannin profile on wheat flour dough rheology. J. Agric. Food Chem..

[22] Wang J., Hou G.G., Liu T., Wang N., Bock J. (2018). GlutoPeak method improvement for gluten aggregation measurement of whole wheat flour. LWT.

[23] Güçbilmez Ç.M., Şahin M., Akçacık A.G., Aydoğan S., Demir B., Hamzaoğlu S., Gür S., Yakışır E. (2019). Evaluation of GlutoPeak test for prediction of bread wheat flour quality, rheological properties and baking performance. J. Cereal Sci..

[24] Rakita S., Dokić L., Dapčević Hadnađev T., Hadnađev M., Torbica A. (2018). Predicting rheological behavior and baking quality of wheat flour using a GlutoPeak test. J. Texture Stud..

[25] Sozer N., Cicerelli L., Heiniö R.-L., Poutanen K. (2014). Effect of wheat bran addition on in vitro starch digestibility, physico-mechanical and sensory properties of biscuits. J. Cereal Sci..

[26] Barros F., Awika J., Rooney L.W. (2014). Effect of molecular weight profile of sorghum proanthocyanidins on resistant starch formation. J. Sci. Food Agric..

[27] Demirkesen I., Campanella O.H., Sumnu G., Sahin S., Hamaker B.R. (2013). A Study on Staling Characteristics of Gluten-Free Breads Prepared with Chestnut and Rice Flours. Food Bioprocess. Technol..

[28] Dubat A., Rosell C.M., Gallagher E. (2016). Mixolab: A New Approach to Rheology.

[29] Wang Q., Li Y., Sun F., Li X., Wang P., Sun J., Zeng J., Wang C., Hu W., Chang J. (2015). Tannins improve dough mixing properties through affecting physicochemical and structural properties of wheat gluten proteins. Food Res. Int..

[30] Al-Foudari M., Sidhu J.S., Alhazza A. (2022). Effect of psyllium husk and wheat mill bran fractions on the microstructure and mixograph characteristics of Arabic bread. Saudi J. Biol. Sci..

[31] Chen J., Fei M., Shi C., Tian J., Sun C., Zhang H., Ma Z., Dong H. (2011). Effect of particle size and addition level of wheat bran on quality of dry white Chinese noodles. J. Cereal Sci..

[32] Chung O., Ohm J., Caley M., Seabourn B. (2001). Prediction of baking characteristics of hard winter wheat flours using computer-analyzed mixograph parameters. Cereal Chem..

[33] Ma F., Baik B.K. (2017). Qualitative effect of added gluten on dough properties and quality of Chinese steamed bread. Cereal Chem..

[34] Gómez M., Ronda F., Blanco C.A., Caballero P.A., Apesteguía A. (2003). Effect of dietary fibre on dough rheology and bread quality. Eur. Food Res. Technol..

